# Analysis of spatial and temporal changes in vegetation cover and its drivers in the Aksu River Basin, China

**DOI:** 10.1038/s41598-024-60575-9

**Published:** 2024-05-03

**Authors:** Yongkang Ding, Yuqing Feng, Kang Chen, Xiaochen Zhang

**Affiliations:** 1 School of Water Resources and Environment, Hebei GEO University, Shijiazhuang, 050031 China; 2 Hebei Province Key Laboratory of Sustained Utilization and Development of Water Resources, Shijiazhuang, 050031 China; 3 Hebei Province Collaborative Innovation Center for Sustainable Utilization of Water Resources and Optimization of Industrial Structure, Shijiazhuang, 050031 China; 4 Hebei Center for Ecological and Environmental Geology Research, Shijiazhuang, 050031 China; 5 Present Address: School of Water Resources and Environment, Hebei GEO University, Huai’an East Road No. 136, Shijiazhuang, 050031 People’s Republic of China

**Keywords:** Aksu River basin, Vegetation cover, NDVI, Geodetector model, Driving factors, Climate-change ecology, Ecological modelling, Hydrology, Ecosystem ecology

## Abstract

Exploring vegetation dynamics in arid areas and their responses to different natural and anthropogenic factors is critical for understanding ecosystems. Based on the monthly MOD13Q1 (250 m) remote sensing data from 2000 to 2019, this study analyzed spatio-temporal changes in vegetation cover in the Aksu River Basin and predicted future change trends using one-dimensional linear regression, the Mann–Kendall test, and the Hurst index. Quantitative assessment of the magnitude of anthropogenic and natural drivers was performed using the Geodetector model. Eleven natural and anthropogenic factors were quantified and analyzed within five time periods. The influence of the driving factors on the changes in the normalized difference vegetation index (NDVI) in each period was calculated and analyzed. Four main results were found. (1) The overall vegetation cover in the region significantly grew from 2000 to 2019. The vegetation cover changes were dominated by expected future improvements, with a Hurst index average of 0.45. (2) Land use type, soil moisture, surface temperature, and potential vapor dispersion were the main drivers of NDVI changes, with annual average *q*-values above 0.2. (3) The driving effect of two-factor interactions was significantly greater than that of single factors, especially land use type interacts with other factors to a greater extent on vegetation cover. (4) The magnitude of the interaction between soil moisture and potential vapor dispersion and the magnitude of the interaction between anthropogenic factors and other factors showed an obvious increasing trend. Current soil moisture and human activities had a positive influence on the growth of vegetation in the area. The findings of this study are important for ecological monitoring and security as well as land desertification control.

## Introduction

Vegetation is a vital component of terrestrial ecosystems and plays a critical role in global material and energy cycles, information transfer, and soil conservation as a key hub connecting the atmosphere, water, and soil^1^. Vegetation growth in arid zones and vegetation cover changes are related to the stability of the regional ecosystem, and the trend of its change is also a key indicator for judging the change in an ecosystem^2^. The analysis of specific watershed areas helps to understand the interaction of driving factors within terrestrial ecosystems and the trend of vegetation responses to ecosystems, thus enabling humans to correctly manage the relationship between ecological environmental protection and socio-economic development^3–5^. Vegetation cover can be characterized by the normalized difference vegetation index (NDVI), and it has been widely used by many scholars^6–9^ to investigate the growth status as well as the trend of vegetation cover^10–13^. Driving factors affecting NDVI changes are mainly divided into natural factors and anthropogenic factors. Previous studies have focused on natural factors, including precipitation and temperature^14–16^, but there is limited research exploring whether potential vapor dispersion and soil moisture significantly affect vegetation growth in arid areas. Under the influence of socio-economic development and urbanization processes, quantitative research on the impact of anthropogenic factors and the interaction between natural and anthropogenic factors is thus required^17,18^.

In the context of global climate change and strong synergies between natural and anthropogenic factors, the growth of regional vegetation has increased dramatically, and the interaction between vegetation and climate change has received special attention from researchers. Previous researchers have utilized a range of diverse analysis methods, such as geographically weighted regression models, which effectively detect heterogeneity within ecosystems by inferring functional relationships between multiple drivers and quantitatively analyzing them. These models are now commonly used to analyze spatial dissimilarities, linear relationships, and correlations. Ren et al.^15^ used such a model to analyze the correlation between vegetation NDVI and natural factors in Jilin Province, China. Yang et al.^19^ used this type of model to assess the spatio-temporal nonlinearity and non-stationarity of climate drivers. With the help of the gray correlation method, the degree of influence between multiple drivers can be calculated using parameters such as the gray correlation coefficient and gray correlation degree. Wang et al.^20^ used this method to quantitatively analyze the relationship between hydrology and vegetation in the Yellow River Basin. Tang et al.^21^ used this method to quantify the response of vegetation cover to ecosystem services in the Jing River Basin on the Loess Plateau. Simulated biophysical process modeling driven by remotely sensed data can integrate biochemical and biophysical processes in terrestrial ecosystems and is often used to analyze the response of communities and a local ecosystem to such influences. Kagan et al.^6^ used this type of model to assess the link between the presence of animals and changes in vegetation in the Judean Hills region of Israel. Mdluli et al.^22^ used this approach to analyze the response of vegetation cover to ecosystem services in the Jing River Basin on the Loess Plateau. Wen et al.^23^ also used the same type of model to clarify the effect of vegetation cover on bird and small mammal community structure in Telperion Nature Reserve, Mpumalanga, South Africa. However, these methods have been typically used to quantitatively assess the impacts of natural factors on vegetation changes, and there are shortcomings and deficiencies in published research on the impacts of human activities. As human activities have become more pronounced, the residual analysis method has been more widely used in quantitative studies of anthropogenic impacts^15,24^. This method calculates the difference between the predicted and true values of the NDVI and evaluates this difference as the magnitude of the statistical anthropogenic influence on vegetation cover change.

Although the above traditional methods can evaluate spatial and temporal changes in vegetation characteristics, they lack quantitative data analysis and delineation of optimal thresholds for factor effects in studies that cover multiple influencing factors simultaneously, as well as for studies on the interaction between pairs of factors. Accordingly, Wang et al.^25^ constructed the Geodetector model to conduct qualitative and quantitative analysis to reveal the strength of driving factors based on the spatial heterogeneity of a study area. The strength of the interaction between two factors and risk area detection can also be calculated by the model to enhance and explore the comprehensiveness of the impact of each factor. So far, the Geodetector model has been discussed and studied in multiple fields of natural and social sciences^26–28^.

The Aksu River Basin is located at the northern edge of the Taklamakan Desert^29^, which is greatly influenced by the desert climate and is one of the sources of wind and sand in Xinjiang, China. As a typical area sensitive to climate change with fragile vegetation ecosystems in China, the ecological quality of vegetation in the Aksu River Basin region has been affected by both climate change and human activities in recent decades^30^. On the other hand, in recent decades, urbanization in the region, mainly in the cities of Aral and Aksu, has promoted the large-scale expansion of construction land. This development has had a profound impact on regional ecosystem services^31^. Ling et al.^32^ found that the growth of groundwater reserves in the Aksu region increased plant cover, while the growth of towns and cities reduced ecological quality, with the type of land use in the region being the most important driver. Yu et al.^33^ found that the ecological environmental quality index of the Aksu region decreased from 0.71 to 0.36 during 2013–2019, and the areas of obvious decreases were concentrated in the settled areas. Previous studies on vegetation change in the Aksu River Basin have had limitations in their designs. For example, some studies have examined only the correlation between vegetation and climate change, ignoring the effects of multiple factors on vegetation growth^33,34^. This may lead to biased conclusions, as climate is not the only factor affecting vegetation growth. In addition, most of the studies have selected fewer types of indicators and have certain limitations. In recent research results, most scholars have considered multi-year averages as a comprehensive measure for inferring drivers of change, while there is limited research quantifying the magnitude and temporal changes in drivers over different time periods^35,36^. Consequently, there is insufficient research on vegetation changes at the watershed scale that accurately estimate the specific magnitude of changes during different time periods under different drivers and their trends. It has not been possible to infer the optimal threshold intervals over which each driver influences vegetation growth during different time periods. As such, the monitoring of soil and water conservation has been insufficiently detailed.

In the present study, utilizing remote sensing data from MODIS and satellite observations, we aimed to (1) analyze the spatial and temporal characteristics of vegetation cover in the Aksu River Basin during 2000–2019 and predict future trends; (2) quantitatively evaluate the magnitude of the driving forces of anthropogenic and natural factors, interactions among the different drivers, and the trends of the changes during five time periods; and (3) detect the optimal threshold range or land use type of major influencing factors favoring vegetation growth in the Aksu River Basin.

## Materials and methods

### Study area

**Figure 1:**
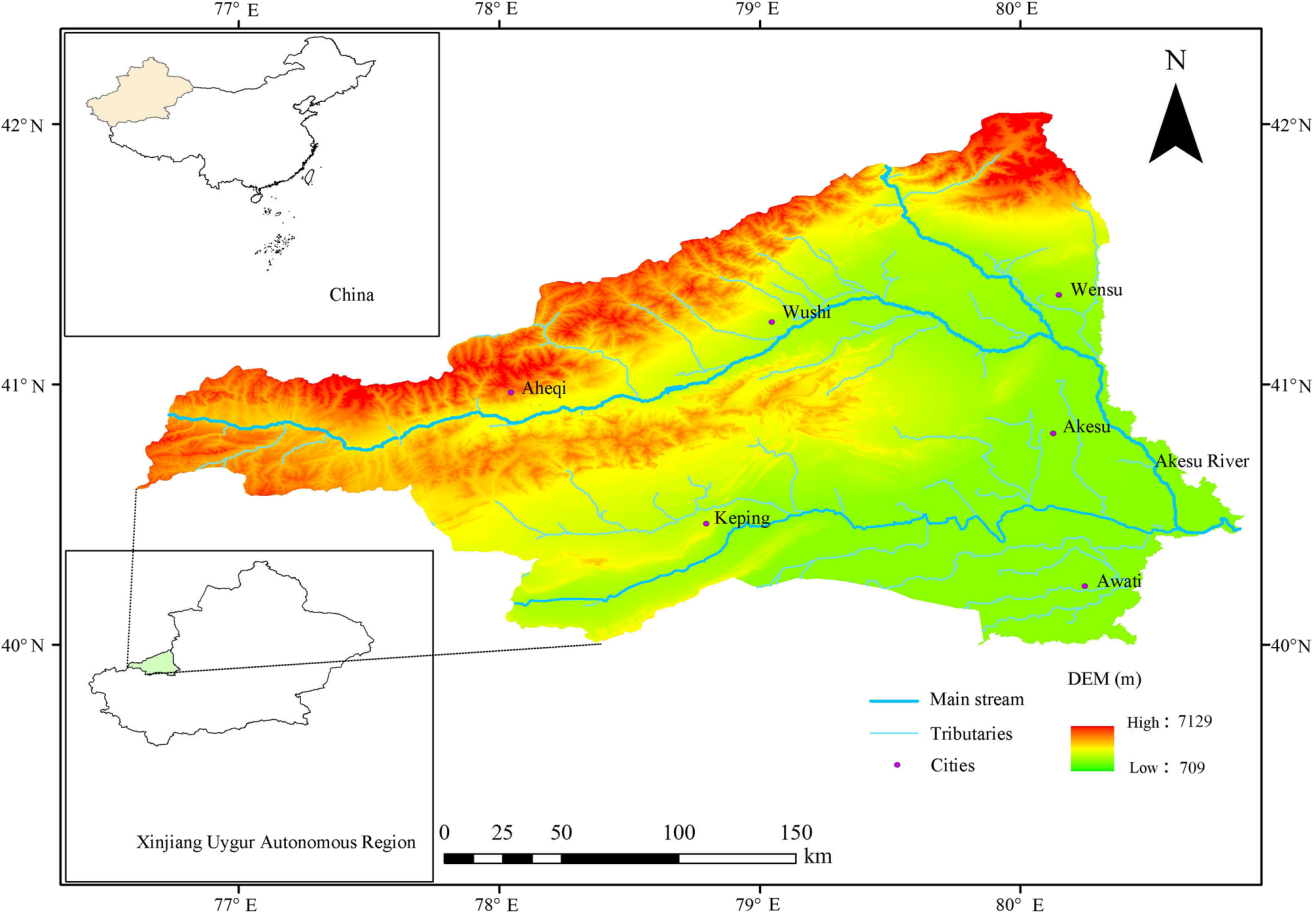
Geographical location of the Aksu River Basin.

Xinjiang Uygur Autonomous Region, located in the northwest of China, is a typical arid region. The Aksu River Basin is situated in the western region of the Tarim Basin inside the Xinjiang Uygur Autonomous Region. The Aksu River is a prominent waterway along the southern border of the Tarim Basin. It enters the territory of China from Kyrgyzstan and subsequently becomes the Aksu River following the confluence of the Kumarak River from the north and the Tashkent River from the west. Eventually, it converges with the Yarkand River and joins the Tarim River in Awati County. The geographical location of the basin is roughly 39°55′–42°6′N, 76°20′–81°10′E, with a watershed area of about 41,147 km^2^ in China (Fig. 1)^37^. The western region of the basin exhibits significant variation in elevation, ranging from 0 to 6140 m above sea level. The basin traverses various administrative divisions, including Ahechi County, Aksu City, Wensu County, Wushi County, Keping County, and Awati County, as well as other townships and settlements. The distribution of precipitation exhibits spatial and temporal disparities^38–40^, wherein mountainous regions serve as primary precipitation zones, experiencing an annual average of 300–450 mm of precipitation. In contrast, the plains of the Aksu River Basin receive a significantly lower annual precipitation total of 50–60 mm^41,42^. Moreover, precipitation within the year is unevenly distributed, being concentrated from June to September. Compounded by its average annual potential evaporation rate of 1000–1500 mm^43,44^, the region faces a relative scarcity of water resources. Consequently, the local population increasingly relies on groundwater reserves. With an average annual temperature of 10–13 °C^45,46^, the vegetation cover in the area is low and sparse under the multiple effects of topography, water, heat, and climate conditions, and human activities, and the degree of land salinization and desertification has increased^47^.

### Data sources

This work utilized NDVI data obtained from the MOD13Q1 dataset, which is available from the NASA website (http://ladsweb.modaps.eosdis.nasa.gov/search/order). The NDVI time series for the geographical regions h23v04 and h24v04, covering the period from 2000 to 2019, was subjected to a series of pre-processing steps. These steps included stitching, projection, cropping, and removal of negative values. Additionally, the interference caused by clouds and atmospheric factors was mitigated using the maximum synthesis method, which effectively captures the spatial and temporal variation in vegetation cover within the specified area.

The ground surface temperature data were obtained from MOD11A1 daytime and nighttime surface temperature data (2000–2019) provided by NASA Earth Data (https://www.earthdata.nasa.gov/) with a temporal resolution of 8 d and a spatial resolution of 1 km after extracting the sub data set followed by the stitching, projection raster, unit conversion, and cropping steps. The annual mean temperature and precipitation data were obtained from the China Meteorological Data Network (http://data.cma.cn), including monthly data from several meteorological stations in the region for the period 2000–2019, and the spatial data were generated by interpolating the annual value series data with a spatial resolution of 1 km using ANUSPLIN interpolation software based on the provided mean monthly precipitation and mean monthly temperature data. Potential vapor dispersion and soil moisture data from 2000 to 2019 with a spatial resolution of about 25 km were obtained from the Global Terrestrial Evapotranspiration Amsterdam Model dataset (https://www.gleam.eu), which comprehensively accounts for forest interception, soil water vapor evapotranspiration, and cloudy conditions that produce outliers.

Data on land use type, vegetation type, soil type, GDP, and population density were obtained from the China Resources and Environment Science and Data Center (http://www.resdc.cn) and downloaded for five periods in 2000, 2005, 2010, 2015, and 2019, respectively. The land use data were obtained from the remote Sensing Monitoring database of land use status in China, with a spatial resolution of 1 km. Vegetation distribution data and soil distribution data were digitally obtained from the 1:100,000 *Vegetation Atlas of China* and the 1:100,000 *Soil Map of China*, respectively. The GDP and population density data were derived from the 1-km grid GDP and the 1-km grid dataset of Chinese population spatial distribution. The Digital Elevation Model (DEM) data were obtained from ASTER GDEM V3 elevation data from the Geospatial Data Cloud Platform (http://www.gscloud.cn), with a spatial resolution of 30 m.

### Methods

#### Trend analysis and significance test

To visually characterize the trend of vegetation cover in the study region, the NDVI values in each image element were combined with remote sensing image data to eliminate outliers^48^, and the trend in vegetation cover in the region between 2000 and 2019 was studied by using one-dimensional linear regression analysis with the following equation^49^.

1 $$ \theta_{slope} = \frac{{n \times \sum\limits_{i = 1}^{n} {\left( {i \times NDVI_{i} } \right)} - \left( {\sum\limits_{i = 1}^{n} i } \right) \times \left( {\sum\limits_{i = 1}^{n} {NDVI_{i} } } \right)}}{{n \times \sum\limits_{i = 1}^{n} {i^{2} } - \left( {\sum\limits_{i = 1}^{n} i } \right)^{2} }} $$

Here, $\theta_{slope}$ is the regression slope of the linear equation; $n$ is the cumulative number of years of the monitoring period ($n$ = 20), and $NDVI_{i}$ is the NDVI value for year $i$. The correlation between NDVI values and time series was used to determine the significant interannual variation of vegetation cover in the area, with a positive slope indicating an increase in vegetation cover and vice versa. Based on previous studies^18,50^, in order to accurately analyze the vegetation change status in the area, the Mann–Kendall trend test was also used to test the significance of NDVI trends, and the significance at the *p* < 0.05 level was determined as follows: − 1.96 ≤ Z ≤ 1.96, non-significant change; Z > 1.96 or Z < − 1.96, significant change. The values were classified into one of five levels by superposition analysis, and the specific criteria are shown in Table 1.

**Table 1:** Classification criteria for vegetation coverage changes based on regression slope of vegetation coverage ($\theta_{slope}$).

Level	$\theta_{{{\text{slope}}}}$	Z
Obvious degradation	≤ − 0.009	< 1.96
Mild degradation	− 0.009 ~ − 0.0009	− 1.96 ~ 1.96
Basically stable	− 0.0009 ~ 0.0009	Arbitrarily
Slight improvement	0.0009 ~ 0.009	− 1.96 ~ 1.96
Significant improvement	≥ 0.009	> 1.96

#### Hurst index

The Hurst index describes how variables depend on time series and indicates their pattern. The calculation of the Hurst index is summarized below.

The series of mean values for any positive integer τ can be described as follows:

2 $$ \overline{{NDVI_{\tau } }} = \frac{1}{\tau } \times \sum\limits_{{{\text{t}} = 1}}^{\tau } {NDVI_{\tau } } $$

$X(t,\tau )$, the cumulative deviation of τ over time, can be described as follows:

3 $$ X(t,\tau ) = \sum\limits_{t = 1}^{\tau } {\left( {NDVI_{t} - \overline{{NDVI_{\tau } }} } \right)} ,1 \le t \le \tau $$

Based on the extreme values of $X(t,\tau )$,$R_{\tau }$ can be calculated as follows:

4 $$ R_{\tau } = \mathop {\max }\limits_{{1 \le {\text{t}} \le \tau }} X\left( {{\text{t,}}\tau } \right) - \mathop {\min }\limits_{{1 \le {\text{t}} \le \tau }} X\left( {{\text{t,}}\tau } \right),\tau = 1,2,3, \cdots ,n $$

The standard deviation $S_{\tau }$ is calculated as follows:

5 $$ S_{\tau } = \left[ {\frac{1}{\tau } \times \sum\limits_{t = 1}^{\tau } {\left( {NDVI_{t} - \overline{{NDVI_{\tau } }} } \right)^{2} } } \right] \times \frac{1}{2},\tau = 1,2,3, \ldots ,n $$

Thus, ${\text{H}}$, the Hurst index can be calculated as follows:

6 $$ \frac{{R_{\tau } }}{{S_{\tau } }} = \left( {\frac{\tau }{2}} \right)^{H} $$

When 0 ≤ *H* < 0.5, NDVI is not persistent; when *H* = 0.5, there is randomness in NDVI changes; when 0.5 < *H* ≤ 1, NDVI is persistent, and the closer *H* is to 1, the stronger the persistence of the phenomenon^51^. The results of the slope trend analysis were overlaid with the Hurst index results to predict the future trend of NDVI over time in the region.

#### Geographical probe calculation

The Geodetector model is a statistical methodology that can be utilized to identify geographical anisotropy, assess the significance and contribution of individual drivers, measure the strength of interactions between components, and facilitate the detection of potential risks. The concept is grounded in the principles of spatial statistics and spatial autocorrelation.

##### Factor detection

Geodetector is used to detect the spatial heterogeneity of the dependent variable *Y* (i.e., NDVI values) and the magnitude of the influence of the independent variable *X* (i.e., selected natural and anthropogenic factors) on the spatial heterogeneity of *Y*, measured by the *q* value^25^.

7 $$ q = 1 - \frac{1}{{N\sigma^{2} }}\sum\limits_{h = 1}^{L} {N_{h} \sigma_{h}^{2} } = 1 - \frac{SSW}{{SST}} $$

8 $$ SSW = \sum\limits_{h = 1}^{L} {N_{h} \sigma_{h}^{2} } $$

9 $$ SST = N\sigma^{2} $$

Here, *h* is the number of categorical terms of the independent variable *X*, ${\text{h}}$ = 1, …, $L$, with a value range of [0, 1], and the larger the value, the stronger the influence of *X* on the spatial differentiation of *Y*; $N_{h}$ and $N$ are the number of cells in category *h* and in the whole region, respectively; $\sigma_{h}^{2}$ and $\sigma^{2}$ are the variance of category ${\text{h}}$ and of the whole region *Y*, respectively; $SSW$ and $SST$ are the sum of the variance of category $L$ and the total variance of the whole region, respectively.

The corresponding *X* and *Y* attribute values were then extracted from the raster data and subsequently utilized in the Geodetector model for computation and analysis. The natural and anthropogenic factors are shown in Table 2, and the driving factors, such as precipitation, air temperature, surface temperature, potential vapor dispersion, soil moisture, elevation, GDP, and population density, were classified into seven categories by the natural breakpoint method. Soil types were classified into 12 categories, vegetation types into eight categories, and land use types into six categories according to the Chinese national criteria for broad categories^50^.

**Table 2:** Classification of natural and anthropogenic factors based on geographic detectors.

Natural factor		Anthropogenic factor	
Precipitation	X1	GDP	X9
Temperature	X2	Population density	X10
Surface temperature	X3	Land use type	X11
Potential vapor dispersion	X4		
Soil moisture	X5		
Soil type	X6		
Vegetation type	X7		
Elevation	X8		

##### Interaction detection

This method can identify the interaction between different independent variables *X* and calculate whether the influence of the two factors on *Y* is related or independent when they act together. The value can be represented by q(X1 ∩ X2), as shown in Table 3.

**Table 3:** Model driving force size criterion of interval and interaction.

Criterion of interval	Criterion of interaction
Min[q(X_1_),q(X_2_)] > q(X_1_ ∩ X_2_)	Nonlinear weakening
Min[q(X_1_), q(X_2_)] < q(X_1_ ∩ X_2_) < Max[q(X_1_),q (X_2_)]	Single-factor nonlinear weakening
Max[q(X_1_),q (X_2_)] < q(X_1_ ∩ X_2_)	Dual factor enhancement
q(X_1_ ∩ X_2_) = q(X_1_) + q(X_2_)	Independence
q(X_1_ ∩ X_2_) > q(X_1_) + q(X_2_)	Nonlinear enhancement

#### Risk area detection

This method is also used to determine whether there is a significant difference in the mean values of attributes between two subregions, and *t*-statistics are used to test the hypothesis that the two subregions differ.

10 $$ t = \frac{{\overline{Y}_{h = 1} - \overline{Y}_{h = 2} }}{{\left[ {\frac{{Var(Y_{h = 1} )}}{{n_{h = 1} }} + \frac{{Var(Y_{h = 2} )}}{{n_{h = 2} }}} \right]^{\frac{1}{2}} }} $$

Here, $\overline{{\text{Y}}}$ is the mean value of the linear regression coefficient of NDVI of vegetation in subregion ${\text{h}}$; ${\text{n}}_{{\text{h}}}$ is the number of samples in subregion ${\text{h}}$; and ${\text{Var}}$ denotes the variance.

## Results

### Analysis of temporal trends in NDVI

**Figure 2:**
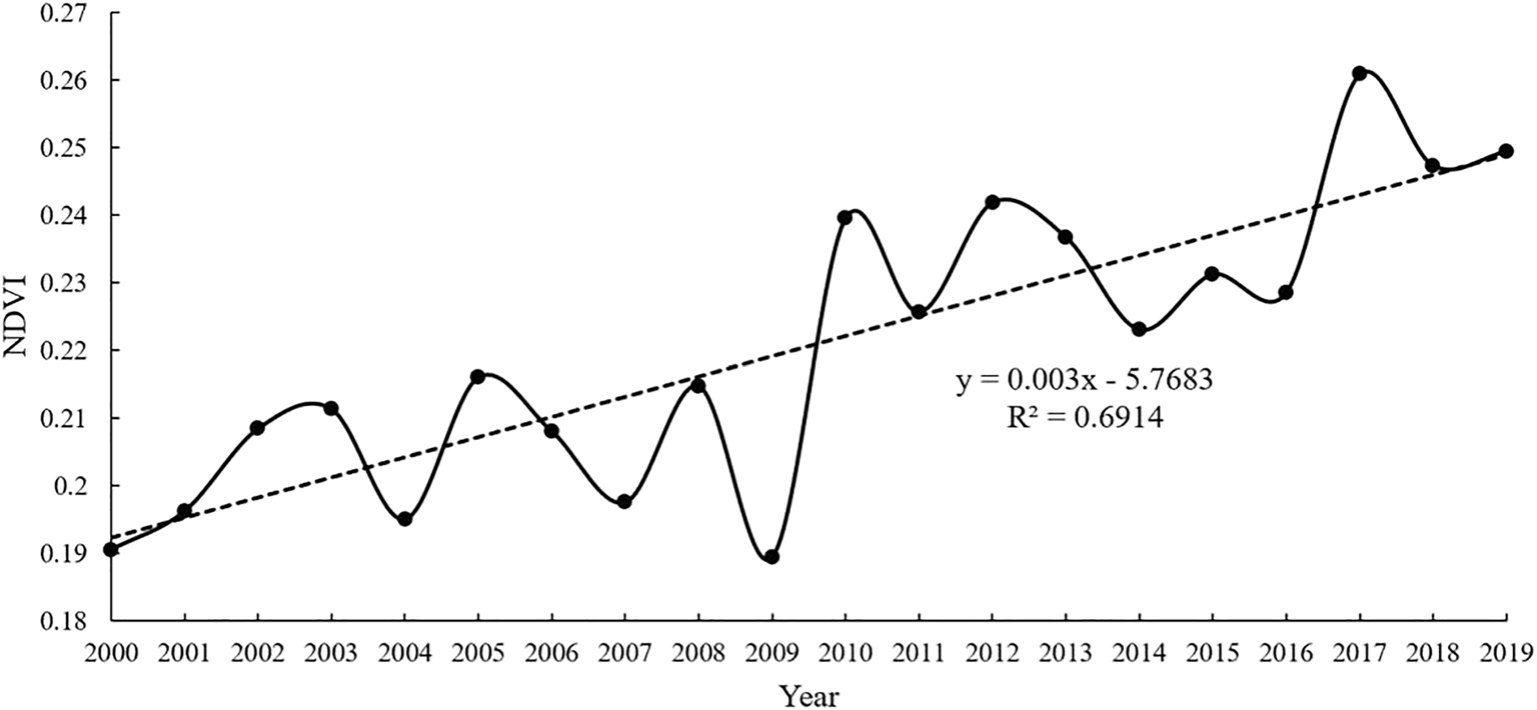
Trend of NDVI from 2000 to 2019.

The annual mean NDVI maximums for each year during 2000–2019 were obtained based on the MVC method utilizing the annual NDVI data (Fig. 2). As shown in Fig. 2, NDVI increased from 0.192 to 0.251, with a mean value of 0.223, a growth percentage of 30.73%, and an annual growth rate of 0.003 a^−1^ during 2000–2019. The analysis showed a significant improvement in the level of vegetation cover and an upward trend in total vegetation. The observed lower value of NDVI was probably related to the stronger summer drought^52^ experienced in 2009.

**Figure 3:**
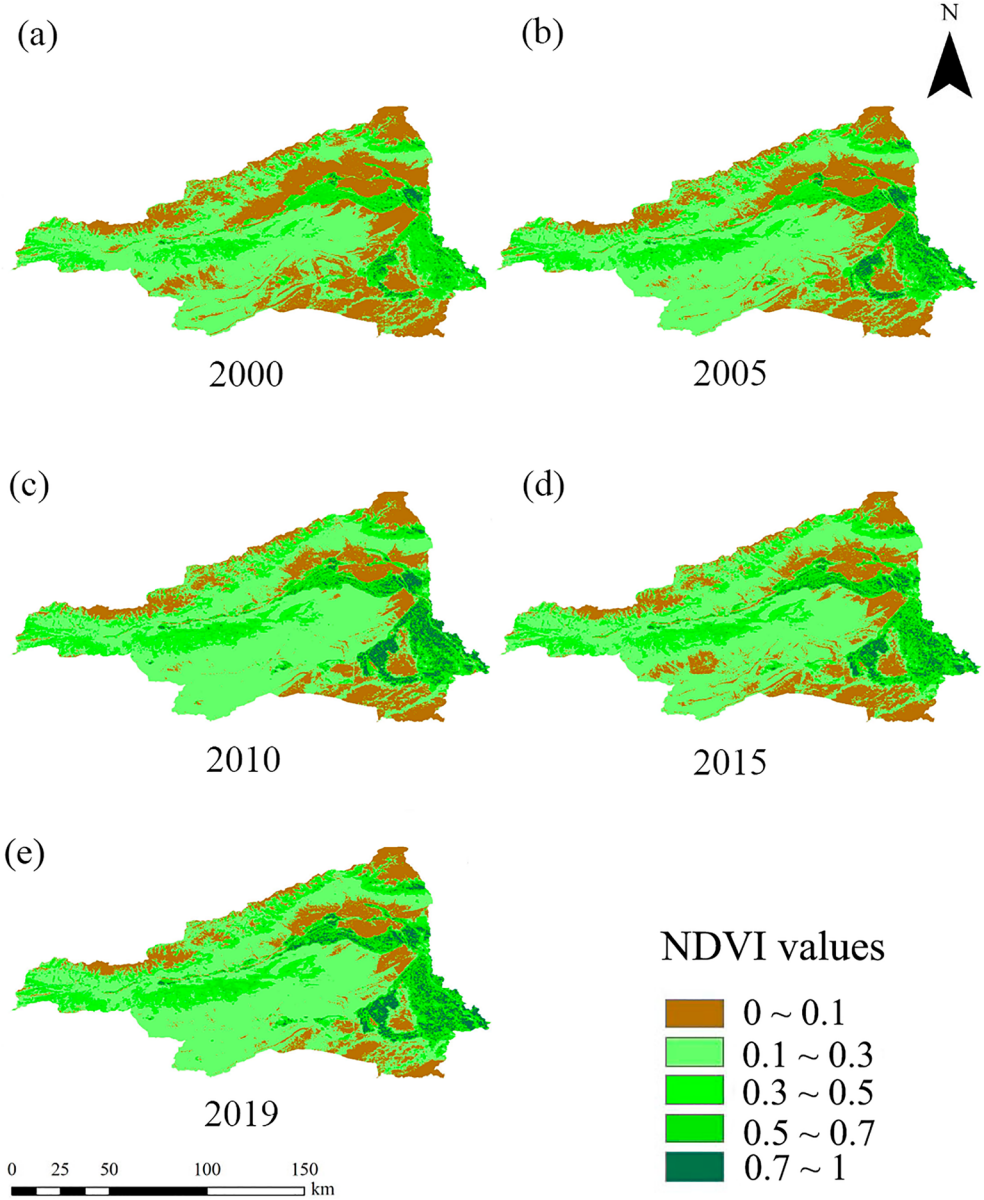
Spatial variation of annual average NDVI zonation from 2000 to 2019.

Owing to its high evapotranspiration and minimal precipitation, the desert areas and the Gobi Desert in particular occupy a large area, and the vegetation cover level is relatively low in this area. The annual maximum NDVI in the region was divided into five classes to investigate the spatial variation in vegetation cover. Referring to existing methods, the vegetation coverage in the area can be classified according to NDVI as follows^18,50^: low vegetation zone (0–0.1), medium–low vegetation zone (0.1–0.3), medium vegetation zone (0.3–0.5), medium–high vegetation zone (0.5–0.7), and high vegetation zone (0.7–1). A vegetation coverage distribution pattern of high in the east and north and low in the west and south can be seen in Fig. 3 for the vegetation in the area, demonstrating clear spatial variability. Low and medium–low vegetation zones predominate in the region’s area; however, medium vegetation zones were primarily spread in the central and western regions, and high and medium–high vegetation zones were primarily concentrated on both banks of the river in the area.

**Figure 4:**
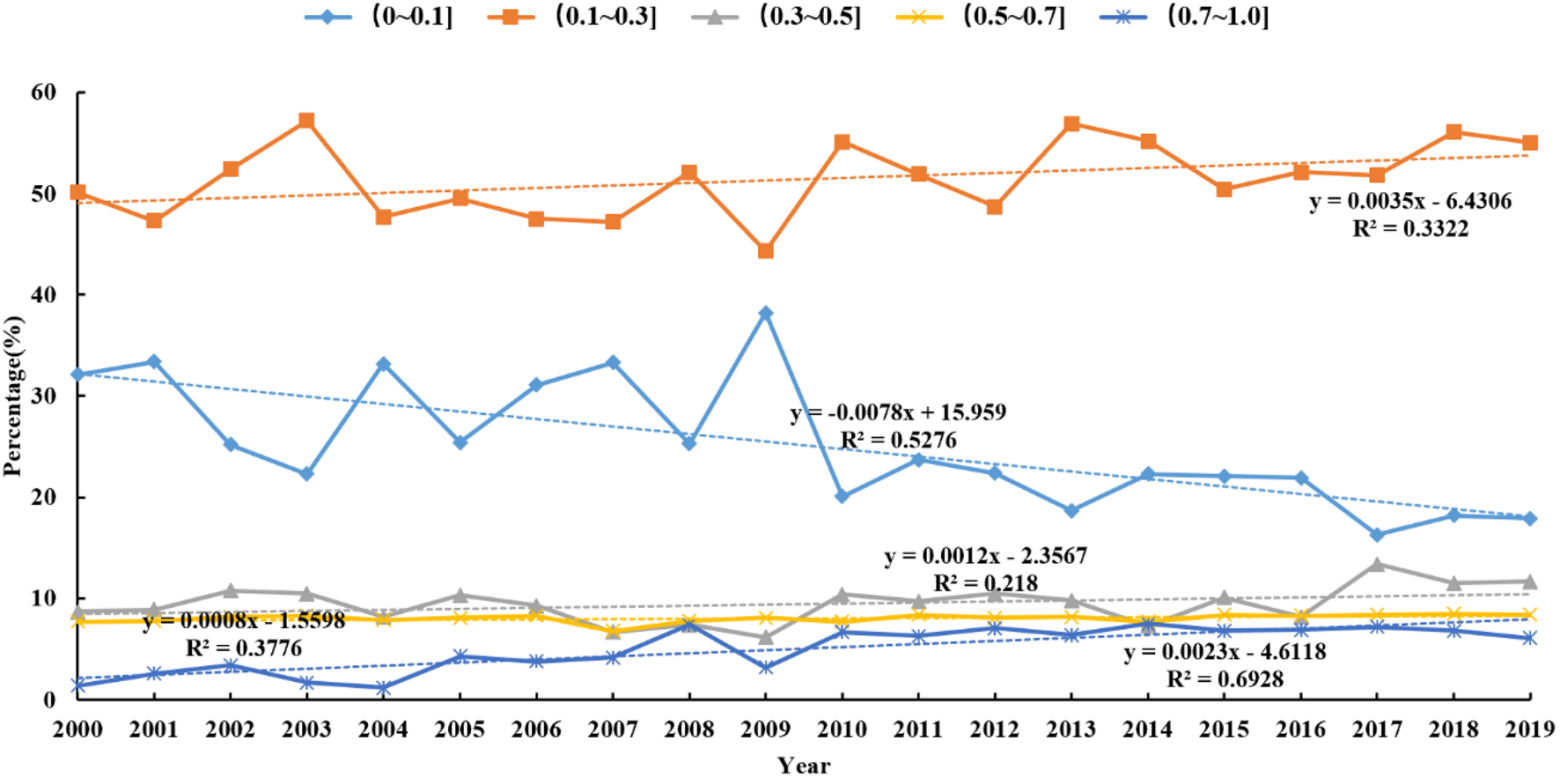
Changes of proportion of NDVI overlay partition of the Aksu River Basin in different years.

As shown in Fig. 4, during the period 2000–2019, the proportion of low and medium–low vegetation zones in the district was large, while the proportion of medium, medium–high, and high vegetation zones was relatively small, and the trend of change was not significant. The annual rate of decrease in the low vegetation zone was − 0.0078 a^−1^, with a decreasing trend in the zone’s share of the whole study area; the rate of increase in the medium–low vegetation zone was 0.0035 a^−1^, and increasing trends were also observed in the medium, medium–high, and high vegetation zones.

The vegetation coverage in the study area in 2000 and 2019 is summarized in Table 4. As shown in Table 4, during the period 2000–2019, the distribution area of the low vegetation zone decreased by 5933 km^2^, accounting for approximately 14.42% of the entire area. On the contrary, there was a significant increase in the coverage area of the other four vegetation types. The increase in vegetation cover zones from low to high coverage was 5.41%, 3.26%, 1.75%, and 4.00%, respectively. This generally indicates that the ecological status of vegetation in the district has tended to improve.

**Table 4:** Changes of NDVI zoning proportion of vegetation in the Aksu River Basin during 2000–2019.

Overlay partition	2000		2019		2000–2019	
	Area (km^2^)	Percentage (%)	Area (km^2^)	Percentage (%)	Area (km^2^)	Percentage (%)
Low vegetation zone	12,961	31.50	7028	17.08	− 5933	− 14.42
Medium–low vegetation zone	20,639	50.16	22,865	55.57	2226	5.41
Medium vegetation zone	3666	8.91	5008	12.17	1342	3.26
Medium–high vegetation zone	3288	7.99	4008	9.74	720	1.75
High vegetation zone	593	1.44	2238	5.44	1645	4.00

### Analysis of spatial trends in NDVI

**Figure 5:**
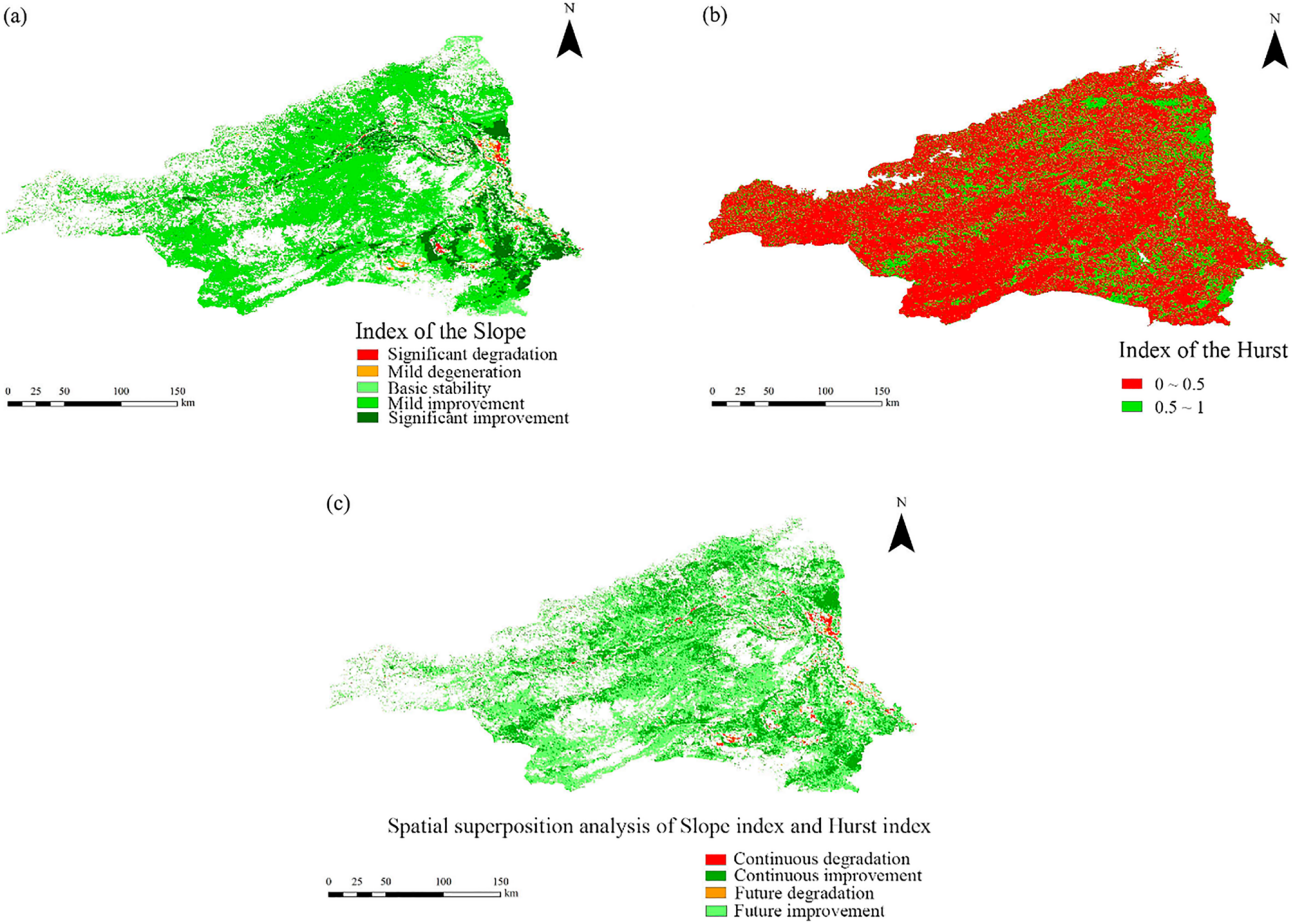
Spatial trends of vegetation cover and Hurst index distribution. (**a**) Trend from 2000 to 2019, (**b**) Hurst index distribution, (**c**) Future trend

One-dimensional linear regression analysis was used to examine the vegetation change in the area between 2000 and 2019. As shown in Fig. 5, the area of vegetation cover in the district that showed improvement is about 30,350.03 km^2^, accounting for approximately 73.76% of the study area. The vast majority of the area showed mild improvement, and the obvious improvement area was mainly distributed in the southeastern part of the study area. However, the area in which the vegetation cover showed degradation covers about 678.92 km^2^, accounting for about 1.65% of the study area. The area of non-significant change spans about 10,118.05 km^2^, accounting for about 24.59% of the study area, though it is unevenly distributed spatially and temporally, being mainly concentrated in the western and northern high-altitude areas, which have been less affected by anthropogenic factors and are therefore less disturbed ecologically. Overall, the mean value of the slope index was 0.004, and the vegetation cover in the area showed a significant trend of improvement during 2000–2019 (Fig. 5a).

The value of the Hurst index in the area ranged from 0.08 to 1, and the average value was 0.45. The area of the study with positive persistent changes in vegetation cover (Hurst > 0.5) accounted for about 25.45% of the study area, while the area of the area with negative persistent change in vegetation cover (Hurst < 0.5) accounted for about 74.55%, indicating that the overall trend of vegetation in the study area was inverse to the current trend (Fig. 5b). The spatial overlay analysis of the Hurst index and slope index shows (Fig. 5c) that the vegetation cover is mainly expected to improve in the future, over 71.51% of the study area, and the area of continuously improved vegetation cover accounts for 26.88%, together occupying most of the area in the district. In contrast, the predicted area of future degradation and continuously degraded vegetation cover account for about 0.82% and 0.79% of the study area, respectively, and these small areas are mainly distributed in the eastern and. The area of future degradation and continuous degradation of vegetation cover accounted for 0.82% and 0.79% of the study area, respectively, mainly in the eastern and southeastern areas, which are relatively densely populated areas.

### Influence of each driver on vegetation cover change

**Figure 6:**
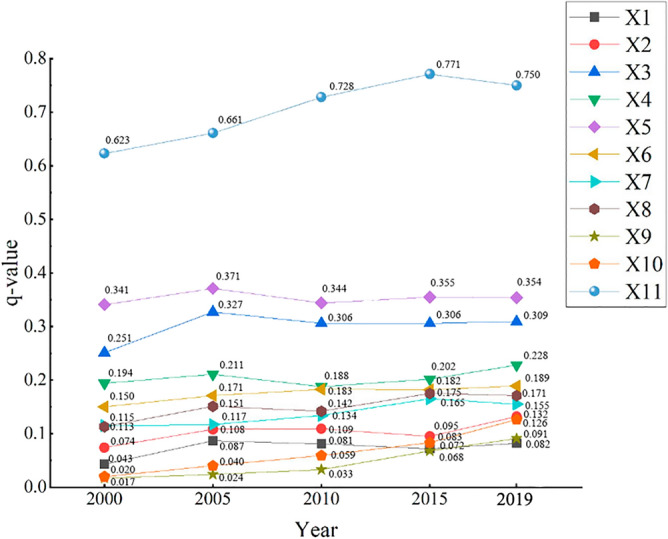
Changes in drivers (q-values) in 2000, 2005, 2010, 2015 and 2019.

Combining a series of datasets on precipitation, air temperature, surface temperature, potential vapor dispersion, soil moisture, soil type, vegetation type, elevation, GDP, population density, and land use type in the study zone for 2000, 2005, 2010, 2015, and 2019, the factor driving force was calculated for each driving factor using the Geodetector model (Fig. 6). Three key results were revealed. (1) The *p*-values corresponding to all driving factors were all less than 0.01, and thus were significant. The dominant factors in all years were uniformly land use type, surface temperature, soil moisture, and potential vapor dispersion, each with *q*-values above 0.2. (2) According to the changes in the *q*-values of each driver during 2000–2019, the overall variation in *q*-values of natural factors was small, with the driver temperature, soil type, vegetation type, and elevation each showing a small increase, while the overall variation in anthropogenic driving factors was large. The *q*-value of GDP increased from 0.017 to 0.091, with a growth rate of 435.29%; the *q*-value of population density increased from 0.020 to 0.126, with a growth rate of 530.00%; and the *q*-value of land use type increased from 0.623 to 0.750, with a growth rate of 20.39%. (3) Factors can be ranked in descending order of their *q*-values in 2000 as follows: land use type, soil moisture, surface temperature, potential vapor dispersion, soil type, vegetation type, elevation, temperature, precipitation, population density, GDP. By 2019, these factors were ranked, quite similarly, in descending order of *q*-values as follows: land use type, soil moisture, surface temperature, potential vapor dispersion, soil type, elevation, vegetation type, temperature, population density, GDP, and precipitation. According to the trend of changes in the last 20 years, the magnitude of *q*-values of population density and GDP in the future are expected to be comparable to the degree of influence of precipitation and temperature among climate factors, indicating that the degree of influence of human activities on vegetation cover changes in the region is increasing and is expected to be more significant in the future.

**Figure 7:**
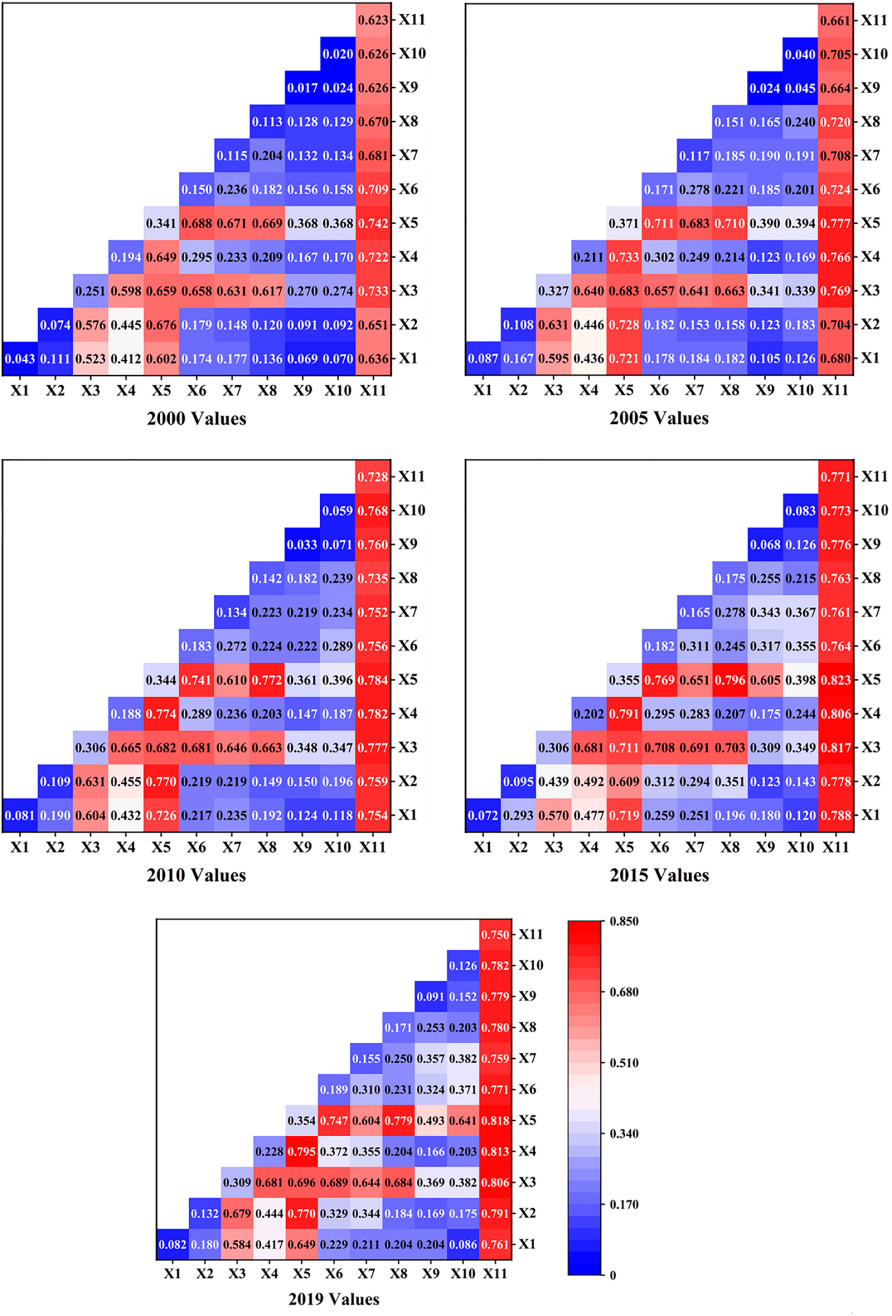
Interactive force of each driver (q-value).

To investigate the influence of NDVI changes under the interaction of natural and anthropogenic factors, interaction drivers were calculated between different drivers for 2000, 2005, 2010, 2015, and 2019 using the Geodetector model. Based on these results, five heat maps were plotted separately using Origin2022 (Fig. 7). (1) The magnitude of the interaction force of any two factors was greater than that of a single factor, and mostly showed non-linear effects. The vegetation growth in the area was not limited to a major single factor, but was the result of the synergistic effect of multiple factors. (2) The interaction between land use type and soil moisture was the most influential in each year (Table 5), with an annual average *q*-value of 0.789, indicating that land use type and soil moisture were the main factors influencing the fluctuation of NDVI values and causing spatial and temporal variation in vegetation cover. (3) The interaction force between land use type and natural factors (soil moisture, potential vapor dispersion, and surface temperature) was significant, with *q*-values greater than 0.7, indicating that under the effect of land use type as the main influencing factor, the interaction with other natural factors had a more significant effect on the change in NDVI. (4) The interaction between soil moisture and potential evapotranspiration increased each year among the natural factors, and the interaction force between them was strongest among the natural factors, showing that changes in vegetation transpiration rate and underlying soil moisture content have a major impact on the NDVI values in the area as a result of the rise in global temperature.

**Table 5:** Interaction force ranking by year (top 4).

Year	Interaction impact ranking (top 4)
2000	X5 ∩ X11(0.742) > X3 ∩ X11(0.733) > X4 ∩ X11(0.722) > X6 ∩ X11(0.709)
2005	X5 ∩ X11(0.777) > X3 ∩ X11(0.769) > X4 ∩ X11(0.766) > X4 ∩ X5(0.733)
2010	X5 ∩ X11(0.784) > X4 ∩ X11(0.782) > X3 ∩ X11(0.777) > X4 ∩ X5(0.774)
2015	X5 ∩ X11(0.823) > X3 ∩ X11(0.817) > X4 ∩ X11(0.806) > X4 ∩ X5(0.791)
2019	X5 ∩ X11(0.818) > X4 ∩ X11(0.813) > X3 ∩ X11(0.806) > X4 ∩ X5(0.795)

By utilizing the analysis of the risk area detector in 2000, 2005, 2010, 2015, and 2019, the optimal multi-year average threshold intervals or types that favor the growth of vegetation in the area were obtained based on the annual average of the factors or the types that occur more frequently within each year (Table 6). When annual precipitation is in the range of 79.42–138.8 mm, the average temperature is in the range of − 2.08–1.35 °C, the surface temperature is in the range of 9.97–13.98 °C, potential vapor dispersion is in the range of 604.59–774.95 mm, soil moisture is in the range of 0.22–0.28 m^3^ m^3^, elevation is in the range of 0–1296 m, GDP is in the range of 560.74–1042.27 yuan km^−2^, population density in the value range of 0–23.12 pop km^−2^, and the area contains cultivated vegetation, hydromorphic soil, and arable land as the main types, the vegetation grows better.

**Table 6:** Type or range of adaptation for the 2000–2019 annual average driver detection indicator.

Evaluation index	Vegetation coverage suitable range (type)	Mean NDVI
Precipitation	79.42 ~ 138.8 (mm)	0.2582
Temperature	− 2.08 ~ 1.35 (℃)	0.2796
Surface temperature	9.97 ~ 13.98 (℃)	0.2588
Potential vapor dispersion	604.59 ~ 774.95 (mm)	0.2771
Soil moisture	0.22 ~ 0.28 (m^3^ m^3^)	0.2797
Soil type	Water into soil	0.2794
Vegetation type	Cultivated vegetation	0.2771
Elevation	0 ~ 1296 (m)	0.2582
GDP	560.74 ~ 1042.27 (yuan km^−2^)	0.3045
Population density	0 ~ 23.12 (pop km^−2^)	0.2582
Land use type	Arable land	0.2662

## Limitations and future research

The Aksu River Basin's spatio-temporal vegetation variation characteristics from 2000 to 2019 were examined. The NDVI values of each year in the region were statistically examined using a combination of remote sensing data, methods such univariate linear regression, the Mann–Kendall trend test, and the Hurst index, and the trend in vegetation cover change over time was clarified. Since there is little discussion on the calculation of the characteristics of future spatial and temporal vegetation change trends in the region, this study superimposed the slope index and Hurst index results to analyze and then predict the future spatial change of vegetation cover trends in the region. Among these findings, the overall trend of vegetation cover change is improving, which is consistent with the results of previous studies on the Xinjiang region and the Aksu watershed^53^, and on the spatial scale, NDVI in the zone showed the tendency of being high in the east and north and low in the west and south, with higher elevation and less human activities in the northern part of the zone, and significant human activities in the eastern region, owing to the implementation of government environmental protection policies, land desertification control, and progress in farming technology. The NDVI of 73.76% of the vegetation areas in the zone showed an increasing trend. Additionally, the future vegetation improvement area still accounted for 71.51%, and the vegetation cover condition in the area has been significantly improved, which also further indicates the increasingly significant influence of human activities on vegetation cover.

Previous studies have mainly focused on precipitation and temperature when considering the influencing factors affecting vegetation cover change, and these studies have been mainly qualitative^54,55^. Bu and Fang studied the spatial and temporal characteristics of NDVI and its influencing factors in this region; they noted that precipitation and temperature in the region had a low degree of influence on NDVI variation^56,57^, but the main influencing factors were not clearly proposed. We found that soil moisture and potential vapor dispersion, which have a strong influence on vegetation in arid zones, have a significant effect. With the help of geographic probes and a number of data types, such as land use type, precipitation, and temperature, we were able to analyze the situation of each driver affecting NDVI changes in the study zone by dividing the data into five time periods. The present study showed that the largest *q*-values were found for land use type among all anthropogenic factors and for soil moisture among all natural factors, such that the magnitude of the interaction between soil moisture and potential vapor dispersion and the magnitude of the interaction between anthropogenic factors and other factors showed an increasing trend. In addition, the NDVI of vegetation in the area was much more responsive to the natural factors soil moisture, surface temperature, and potential vapor dispersion and the anthropogenic factor land use type compared to other factors. According to a recent study^58^, moisture is one of the main factors limiting the growth and development of vegetation in the area. The region is located in an arid zone with very little precipitation and high evaporation, the water table in the plains is shallow, and glacial melt, shallow groundwater recharge of soil moisture, and anthropogenic agricultural irrigation can improve soil moisture conditions in the region and provide a good environment for vegetation growth^59^. The zone is dominated by desert vegetation, which is more sensitive to soil moisture content^60^. The eastern plain region has high vegetation cover in some areas, with hydromorphic and semihydromorphic soil, which has an important influence on the spatial and temporal distribution pattern and stability of vegetation. As the types of land use most significantly affected by human activities in the region^61,62^, long-term fertilization and irrigation and land reclamation can, to a certain extent, reduce the adverse effects of climate, topography, and soil infertile on vegetation crops and provide good conditions for vegetation growth and development. Secondly, as one of the major global cotton production areas, the Aksu River Basin has experienced a rapid increase in the proportion of cotton cultivation, and the use of drip irrigation in the Aksu region has been greatly influenced by the selection of human cultivation areas and the implementation of government policies; thus, the impact of human activities on the inter-annual changes in NDVI within the basin should not be ignored^63^.

The current quantitative research and analysis methods of human activities are insufficient, and the Geodetector model, as a popular method for quantitative analysis of human activities, has the advantage of being able to conduct specific quantitative classification of various drivers and determine the weight of each driver, unlike other methods. However, the classification of anthropogenic factors affecting NDVI changes is not detailed enough, and there is a lack of data in published studies, which have mainly considered land use type, population density, and GDP as the main factors. Additional factors, such as policy factors, industrial production and processing pollution, and public awareness of environmental protection, cannot be studied quantitatively. Although the Geodetector model can specifically delineate and calculate the magnitude of driving forces for each factor, the selection of factors and delineation based on the natural interval method are more subjective and thus may not accurately capture all the driving factors and the best-controlled intervals that affect vegetation cover change. Thus, this study collected as much data as possible on various types of drivers in the region; the data based on national broad criteria were strictly classified according to their data types, while the rest of the data were unified for the classification levels to ensure the consistency and accuracy of the data.

Although the overall vegetation coverage in the area was not high, the trend of its improvement was very obvious and consistent with the continuous promotion of relevant government policies. Along with global climate change, ecosystem security in the region continues to face key challenges. Long-term field surveys and ecological monitoring of areas with low vegetation and areas of continued future degradation are still needed to provide a scientific basis for ecological management and the rational use of ecological resources in watersheds.

## Conclusions

In the present study, we assessed the spatial and temporal various and future trends of vegetation in the Aksu River Basin based on one-way linear regression, the Mann–Kendall trend test, and the Hurst index. Then, the Geodetector model was used to reveal the magnitude (*q*-value) of the driving force between NDVI and the driving factors over five time periods. Finally, we predicted the future changes of vegetation cover in the region, derived the year-to-year trends in the response of each driver to NDVI, and delineated the optimal multi-year average threshold interval or land use type that favors vegetation growth. The main findings are as follows.

(1) The vegetation cover in the zone showed a significant increasing trend, with a mean NDVI value of 0.223 and a growth rate of 30.73% at 0.003 a^−1^ during 2000–2019. As a typical arid region, the Aksu River Basin showed a change of its NDVI that was closely related to the progress of agricultural technology and the implementation of environmental protection policies in the region. (2) The average value of the slope index was 0.004, while the areas of improved NDVI and degradation were 73.76% and 1.65% of the study area, respectively; the average value of the Hurst index was 0.45, and the trend in vegetation change was reversed and continuous. The spatial superposition analysis of the two indices showed that the future vegetation is expected to be dominated by the improvement in conditions, over 71.51% of the study area; in contrast, the future degraded area and the continuous degraded area accounted for just 0.82% and 0.76% of the study area, mainly distributed in densely populated areas. (3) Land use type was the main driver, with an average annual *q*-value of 0.707, followed by drivers such as soil moisture, surface temperature, and potential vapor dispersion. The driving force of anthropogenic factors increased each year during the period and was comparable to that of climatic factors such as precipitation and temperature. This predicts an increasing relationship between human activities and vegetation change and ecosystem trends in the region. (4) The interaction between land use type and soil moisture was the largest, with an annual average *q*-value of 0.789, indicating that land use type and soil moisture were the main factors influencing the fluctuation of NDVI values, leading to spatial and temporal changes in vegetation cover. (5) Based on the risk area detection results, the Geodetector model determined the most areas conducive areas to vegetation growth to be characterized as follows: annual precipitation, 79.42–138.8 mm; average temperature, − 2.08–1.35 °C; surface temperature, 9.97–13.98 °C; potential vapor dispersion, 604.59–774.95 mm; soil moisture, 0.22–0.28 m^3^ m^3^; elevation, 0–1296 m; GDP, 560.74–1042.27 yuan km^−2^; population density, 0–23.12 pop km^−2^; and cultivated vegetation, hydromorphic soils, or arable land as the main types of areas.

## Data Availability

The datasets used and/or analysed during the current study available from the corresponding author on reasonable request.
